# Matricellular protein CCN1 mediates doxorubicin-induced cardiomyopathy in mice

**DOI:** 10.18632/oncotarget.9162

**Published:** 2016-05-04

**Authors:** Pei-Ling Hsu, Fan-E Mo

**Affiliations:** ^1^ Institute of Basic Medical Sciences, College of Medicine, National Cheng Kung University, Tainan, Taiwan; ^2^ Department of Cell Biology and Anatomy, College of Medicine, National Cheng Kung University, Tainan, Taiwan

**Keywords:** CCN1, doxorubicin, cardiotoxicity, integrins, XIAP

## Abstract

Doxorubicin (DOX) is an effective chemotherapeutic agent however its clinical use is limited by its cumulative cardiotoxicity. Matricellular protein CCN1 mediates work-overload-induced cardiac injury. We aimed to assess the role of CCN1 in DOX-associated cardiomyopathy. Here we discovered CCN1 expression in the myocardium 1 day after DOX treatment (15 mg/kg; i.p.) in mice. Whereas CCN1 synergizes with Fas ligand (FasL) to induce cardiomyocyte apoptosis, we found that FasL was also induced by DOX in the heart. To assess the function of CCN1 *in vivo*, knockin mice (*Ccn1^dm/dm^*) expressing an β_6_β_1_-binding defective CCN1 mutant were treated with a single dose of DOX (15 mg/kg; i.p.). Compared with wild-type mice, *Ccn1^dm/dm^* mice were resistant to DOX-induced cardiac injury and dysfunction 14 days after injection. Using rat cardiomyoblast H9c2 cells, we demonstrated that DOX induced reactive oxygen species accumulation to upregulate CCN1 and FasL expression. CCN1 mediated DOX cardiotoxicity by engaging integrin β_6_β_1_ to promote p38 mitogen-activated protein kinase activation and the release of mitochondrial Smac and HtrA2 to cytosol, thereby counteracting the inhibition of XIAP and facilitating apoptosis. In summary, CCN1 critically mediates DOX-induced cardiotoxicity. Disrupting CCN1/β_6_β_1_ engagement abolishes DOX-associated cardiomyopathy in mice.

## INTRODUCTION

Doxorubicin (DOX) is an anthracycline antineoplastic drug to treat a variety of solid tumors and hematological malignancies [1]. The antitumor activity of DOX is attributable to its ability to intercalate DNA helix and inhibit topoisomerase II, causing DNA double strand breaks, G2 arrest and apoptosis in replicating cells [2]. Despite its effectiveness against cancer, the clinical use of DOX is critically limited by its cumulative cardiotoxicity [3]. A greater incidence of DOX-related congestive heart failure was found in patients after receiving a cumulative dose of 400 mg/m^2^ [4]. The onset of cardiac complications can occur during the treatment or up to 10 years after the cessation of DOX therapy [5]. Cardiovascular-related disease derived from the adverse effects of cancer treatments has become the leading noncancer-related cause of morbidity and mortality in long-term cancer survivors [5].

Cardiotoxic effects of DOX, distinct from its antitumor activities, develop from increasing mitochondrial iron content and cellular reactive oxygen species (ROS) levels [6]. Currently, dexrazoxane is the only agent approved by the United States FDA and the European Medicines Agency to prevent long-term cardiotoxicity caused by DOX. Dexrazoxane, structurally similar to EDTA, is a strong chelator of iron and prevents the iron-dependent ROS production induced by DOX [7]. Because ROS also contribute to the antineoplastic activity of DOX [1], dexrazoxane may interfere with the anticancer activity of DOX and leads to the higher latent risk for acute myeloid leukemia and other secondary malignancies in pediatric patients receiving dexrazoxane [8]. A more specific strategy needs to be developed to prevent DOX cardiotoxicity.

Fas ligand (FasL) is a critical mediator in DOX-associated cardiotoxicity [9]. Despite the pivotal role of FasL and its receptor Fas in cardiac injury, the activation of Fas signaling alone is not sufficient to induce apoptosis in mouse hearts or in cultured cardiomyocytes [10, 11]. CCN1, a matricellular protein, sensitizes cardiomyocytes to FasL-induced apoptosis both in mice and in cultured cells [12, 13]. CCN1 promotes various cell activities and regulates cell survival and death through interaction with distinct integrin receptors in a cell type- and context-dependent manner [14]. CCN1 is dynamically expressed in the cardiovascular system during embryogenesis and *Ccn1*-null mice die prenatally from cardiovascular defects [15, 16]. CCN1 expression during adulthood is largely silenced, but is redeployed upon tissue injury and in a variety of pathological conditions [14]. In the heart, CCN1 is induced in end-stage ischemic cardiomyopathy in humans and after myocardial infarction in animal models [17]. CCN1 is also induced in mice with work-overload-induced cardiac injury, and sensitizes FasL-mediated cardiomyocyte apoptosis by engaging its cell-surface receptor integrin α_6_β_1_ [12]. CCN1/α_6_β_1_ signaling triggers the accumulation of cellular ROS, activation of p38 mitogen-activated protein kinase (MAPK), and translocation of mitochondrial Smac and HtrA2 to cytosol, thereby counteracting the anti-apoptotic activity of XIAP and facilitating FasL-induced apoptosis in cardiomyocytes [13].

Because CCN1 is induced upon various cardiac insults to promote cell death, we hypothesized that CCN1 may mediate DOX-associated cardiotoxicity through engaging integrin α_6_β_1_. To test this hypothesis, we first examined the cardiac expression of CCN1 after DOX treatment in mice. Subsequently, a previously generated knockin mice (*Ccn1^dm/dm^*), expressing an α_6_β_1_-binding defective CCN1 mutant with two of its binding sites mutated (double mutant) [18], were used to assess the role of CCN1 in mediating DOX cardiotoxicity. Furthermore, cardiomyoblast H9c2 cells were used for *in vitro* mechanistic studies.

## RESULTS

### CCN1 critically mediates DOX-associated cardiomyopathy

**Figure 1:**
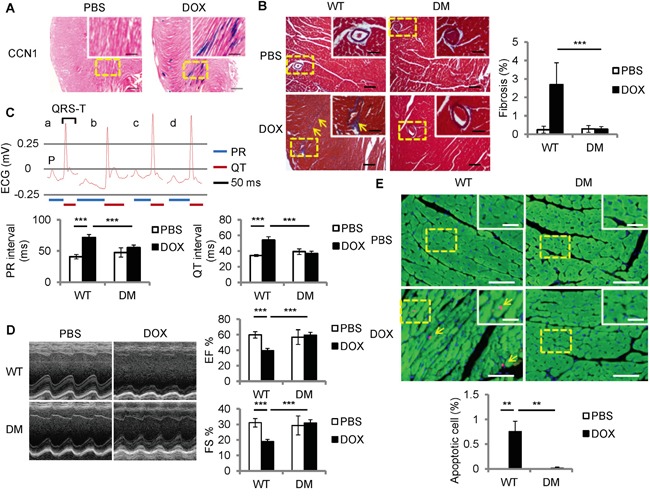
*Ccn1^dm/dm^* mice were resistant to Doxorubicin (DOX)-induced cardiomyopathy **A.** For *Ccn1* expression, the hearts from *Ccn1^+/lacZ^* mice 1 day after DOX treatment (15 mg/kg; i.p.) were stained with X-gal (blue). High power views of the dashed areas were shown in the insets. **B, E.** Cardiac tissue was collected from WT or *Ccn1^dm/dm^* (DM) mice 14 days after PBS or DOX administration (15 mg/kg; i.p.) (*n* = 6 for each group). (B) Cardiac fibrotic lesions were identified by Masson's trichrome staining (blue, arrows). The percentage of the fibrotic area was quantified using the NIH ImageJ program. Data are means ± SEM from 8 comparable tissue sections per mouse. **C, D.** Cardiac physiology was assessed by electrocardiography (ECG) and echocardiography on WT or *Ccn1^dm/dm^* mice (n=4)14 days after PBS or DOX administration. (C) The lengths of PR interval and QT interval were indicated below the representative ECGs from a: WT mice/PBS; b: WT mice/DOX; c: *Ccn1^dm/dm^* mice/PBS; d: *Ccn1^dm/dm^* mice/DOX. Data are means ± SEM from 3 measurements per mouse. (D) Representative echocardiograms demonstrate the structural dynamics during left ventricular systole. Ejection fraction (EF) was computed from the measurements of the end-diastolic volume and end-systolic volume. Fractional shortening (FS) is the fraction of the diastolic dimension that is reduced in systole. Data are means ± SEM from 3 measurements per mouse. (E) The percentage of apoptotic (TUNEL staining, pink nuclei) cardiomyocytes (troponin I^+^ cells, green) indicated by arrows was quantified from 10 random high-power views per tissue section and 8 sections per mouse. Tissue sections were counterstained with DAPI for nuclei (blue). Bars in (A): 500 μm; in the insets of (A): 200 μm. Bars in (B): 100 μm; in the insets of (B): 50 μm. Bars in (E): 50 μm; in the insets of (E): 20 μm.

To examine *Ccn1* expression in DOX-associated cardiomyopathy, DOX was delivered to *Ccn1^+/lacZ^* mice in which an allele of *Ccn1* was replaced by a *lacZ* reporter gene [16]. Heart tissue was collected 1 day after DOX injection (15 mg/kg; i.p.). *Ccn1* expression was assessed through measuring the enzymatic activities of LacZ by X-gal staining. *Ccn1* expression was not detected in the PBS-controlled hearts (Figure 1A). *Ccn1* expression became evident (blue) in the cardiomyocytes 1 day after DOX treatment before any tissue lesions occurred (Figure 1A). To assess the role of CCN1 in mediating DOX cardiotoxicity, DOX was delivered to *Ccn1^dm/dm^* (DM) mice. Heart tissue was collected 14 days after DOX injection. Cardiac fibrotic lesions (blue) identified by trichrome staining were increased in the perivascular areas in the myocardium of wild-type (WT) mice receiving DOX (Figure 1B). DOX-induced fibrotic lesions were not observed in *Ccn1^dm/dm^* mice (Figure 1B). TUNEL^+^ apoptotic cardiomyocytes (green troponin-I^+^ cells with pink nuclei, arrows in Figure 1E) were increased by DOX in the myocardium of WT mice, and were not affected by DOX in *Ccn1^dm/dm^* mice (Figure 1E). The cardiac lesion created by DOX leads to deterioration of cardiac function. Prolonged electrocardiographic (ECG) PR and QT intervals were detected in the WT mice receiving DOX treatment. The ECG measurements were not altered by DOX in *Ccn1^dm/dm^* mice (Figure 1C). Consistently, left ventricular systolic function indices, ejection fraction (EF) and fractional shortening (FS) determined by echocardiography, were better maintained in *Ccn1^dm/dm^* mice after DOX treatment (Figure 1D). Together, these results demonstrated that CCN1 critically mediates DOX-associated cardiotoxicity. Disrupting the binding between CCN1 and integrin α_6_β_1_ effectively prevents the cardiotoxicity of DOX in mice.

### FasL is induced by DOX in cardiomyocytes

**Figure 2:**
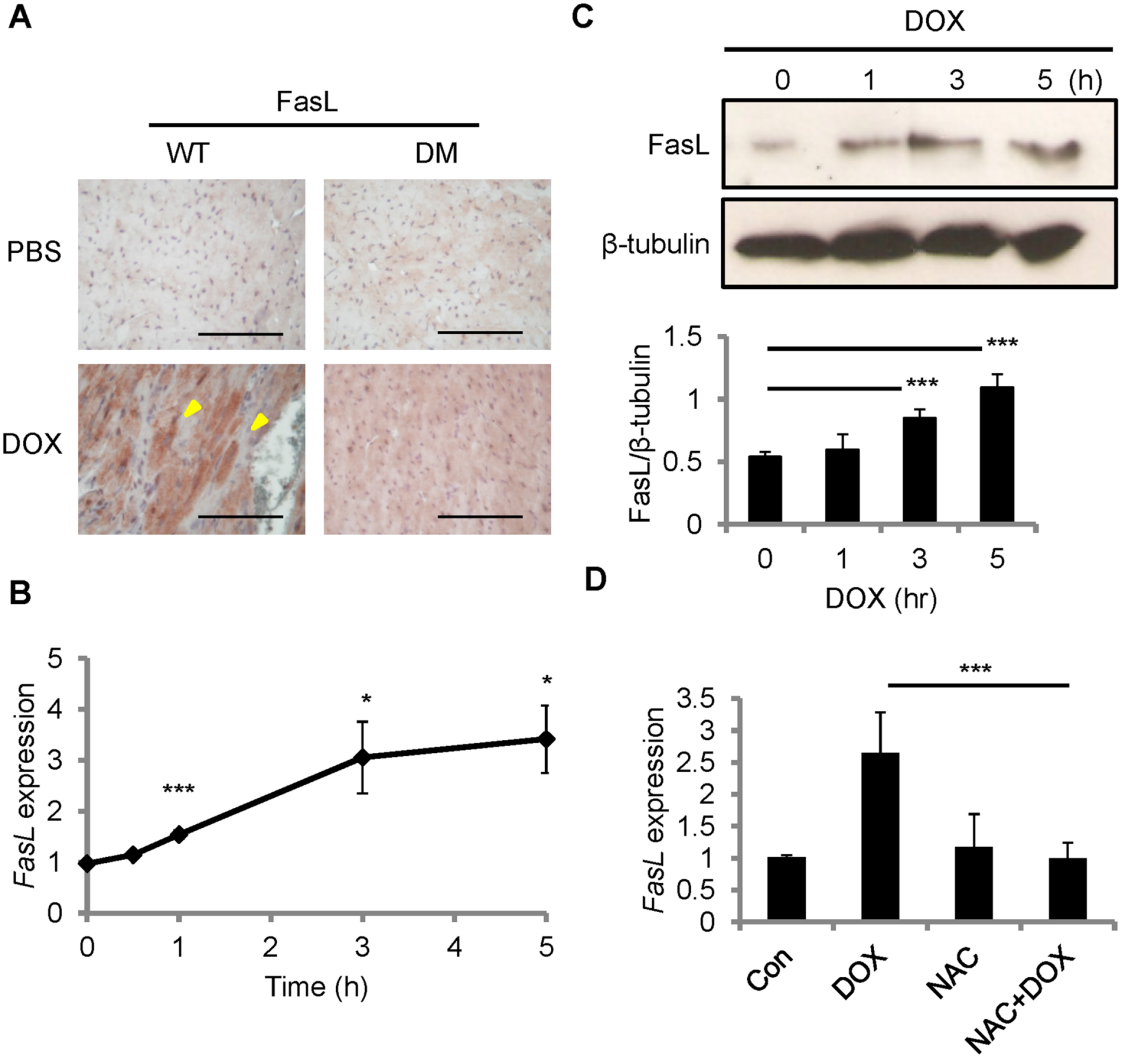
FasL was induced by DOX in the mouse heart and in cardiomyoblast H9c2 cells **A.** Heart tissue sections from WT and *Ccn1^dm/dm^* mice 14 day after PBS or DOX treatments (15 mg/kg; i.p.) were immunohistochemically stained with anti-FasL antibody (reddish-brown). **B, C.** H9c2 cells were treated with DOX (10 μM) for the indicated times before total RNA were isolated for *FasL* qRT-PCR (B), or total cell lysates were isolated and immunoblotted with anti-FasL antibody or anti-β-tubulin antibody as a loading control (C). Protein band intensity was quantified using the ImageJ program. The statistical significance was compared between experimental and control (0 h) groups. **D.** Where indicated, cells were pretreated with ROS scavenger NAC (5 mM) for 30 min prior to adding DOX (10 μM) and culturing for additional 5 h. Subsequently, total RNA was isolated to measure *FasL* mRNA levels by qRT-PCR. Data are expressed as mean ± SEM of triplicate experiments. Bars in (A): 100 μm.

Fas signaling is required for DOX-induced cardiomyopathy in rats [19]. CCN1 promotes cardiomyocyte apoptosis through potentiating the death effect of FasL [12, 13]. We examined the induction of FasL after DOX treatment by immunohistochemical staining with anti-FasL antibody. FasL was increased in the myocardium of WT mice 14 days after DOX injection (reddish-brown staining in Figure 2A). In *Ccn1^dm/dm^* mice, FasL staining was significantly higher in DOX-treated hearts than in PBS controls, though the levels of FasL induction were lower than in WT mice (Figure 2A). To investigate whether DOX directly induces FasL expression in cardiomyocytes, we treated rat cardiomyoblast H9c2 cells with DOX *in vitro*. *FasL* mRNA levels, determined by quantitative real-time PCR (qRT-PCR), were elevated within 1 h and sustained at higher levels from 3-5 h after DOX treatment (10 μM) in H9c2 cells (Figure 2B). Consistently, FasL protein in H9c2 cells, assessed by Western blotting, was increased significantly 3 h after DOX treatment (Figure 2C). Because oxidative stress generated by DOX mediates its cardiotoxicity [20], we tested the requirement of ROS for FasL induction by combining DOX treatment with ROS-scavenger N-acetyl-L-cysteine (NAC). Cotreatment of NAC (10 mM) completely abolished the induction of *FasL* expression by DOX (10 μM; 5 h) (Figure 2D), indicating the requirement of ROS in the upregulation of *FasL* expression.

### CCN1 is induced by DOX in cardiomyocytes *in vitro*

**Figure 3:**
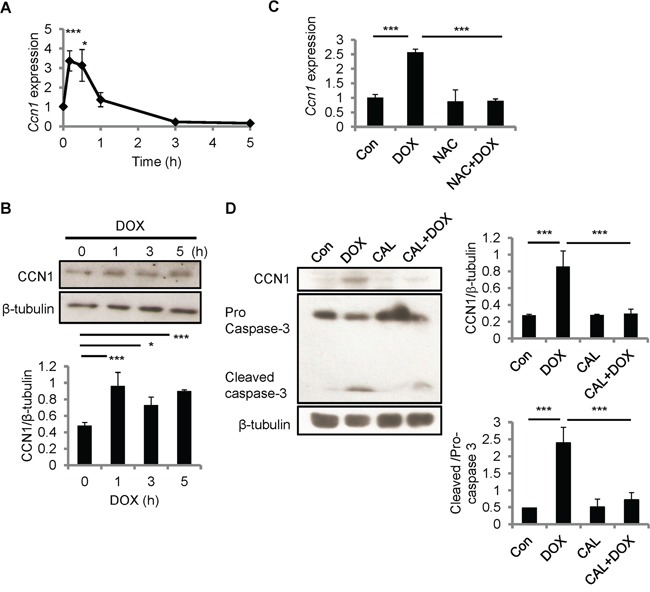
CCN1 was induced by DOX in H9c2 cells **A, B.** H9c2 cells were treated with DOX (10 μM) for the indicated times before total RNA were isolated for *Ccn1* qRT-PCR (A), or total cell lysates were isolated and immunoblotted with anti-CCN1 or anti-β-tubulin antibodies (B). **C.** Where indicated, cells were pretreated with NAC (5 mM) for 30 min prior to adding DOX (10 μM) and culturing for additional 5 h. Subsequently, total RNA was isolated to measure *Ccn1* mRNA levels by qRT-PCR. **D.** Total cell lysates from cells treated with protein kinase C inhibitor calphostin C (1 μM, 1-h pretreatment; CAL) and/or DOX (10 μM, 5 h) were immunoblotted with anti-CCN1, anti-caspase 3, or anti-β-tubulin antibodies. Protein band intensity was quantified using the ImageJ program. All data are expressed as mean ± SEM of triplicate experiments.

Because CCN1 can sensitize cardiomyocytes to FasL-induced apoptosis, we examined CCN1expression in H9c2 cells after DOX treatment using qRT-PCR and Western blotting. *Ccn1* gene expression was induced within 10 min after DOX treatment (10 μM), and tapered off after 1 h (Figure 3A). CCN1 protein levels were elevated 1 h after DOX treatment and the levels sustained through the 5 h treatment period (Figure 3B). *Ccn1* gene expression induced by DOX (10 μM; 30 min) was completely abolished by NAC (10 mM) (Figure 3C), indicating the requirement of ROS in the CCN1 induction by DOX. It has been indicated that protein kinase C (PKC) can be activated by cellular oxidants [21] and PKC activity is essential for stress-induced myocardial CCN1 expression [17]. Here, DOX-induced CCN1 expression and caspase-3 activation (cleavage) in H9c2 cells were completely blocked by PKC inhibitor calphostin C (1 μM, 1-h pretreatment; CAL) (Figure 3D), demonstrating that PKC is required for DOX-induced CCN1 expression and cell apoptosis.

### CCN1 facilitates DOX-induced apoptosis through diminishing XIAP

**Figure 4:**
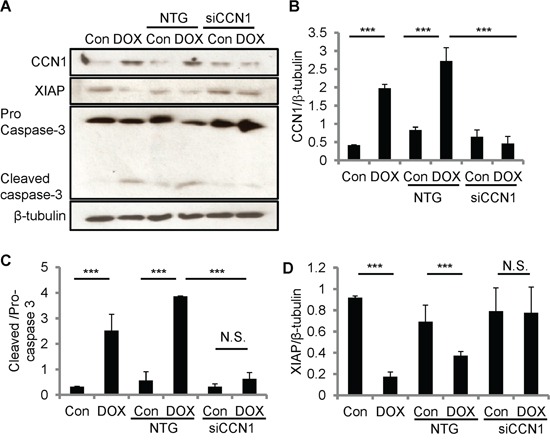
CCN1 critically mediated DOX cardiotoxicity through diminishing XIAP in H9c2 cells The expression of CCN1 was knocked down by *Ccn1* siRNA (siCCN1) or a non-targeting (NTG) control. Transfected cells were cultured for 48 h before further treatments. Cells with or no *Ccn1*-knockdown were treated with or no DOX (10 μM) for 5 h before total cell lysates were harvested and immunoblotted with anti-CCN1, anti-XIAP, anti-caspase 3, or anti-β-tubulin antibodies **A.** Protein band intensity was quantified using the ImageJ program. **B.** Reduced CCN1 band intensities in siCCN1-transfected cells demonstrated the knockdown efficiency. **C.** The ratio of pro-/cleaved-caspase 3 represents the level of caspase-3 activation. **D.** XIAP reduction by DOX was blunted by *Ccn1* knockdown. All data are expressed as mean ± SEM from 3 independent experiments.

To test the role of CCN1 in DOX-induced apoptosis, we knocked down *Ccn1* expression by transfecting H9c2 cells with *Ccn1* siRNA (siCCN1) or non-targeting (NTG) control siRNA 48 h prior to DOX treatment. The levels of CCN1 protein in siCCN1-transfected cells were largely diminished even after DOX treatment (10 μM; 5 h); whereas the control NTG-cells expressed CCN1 upon DOX treatment at the levels similar to nontransfected cells (Figure 4A, 4B). The activation of caspase-3 (cleaved caspases-3) was higher in nontransfected or NTG-cells after DOX treatment (Figure 4A, 4C). By contrast, the activation of caspase-3 was not changed by DOX in siCCN1-cells (Figure 4A, 4C), demonstrating that CCN1 is essential for apoptosis induced by DOX in cardiomyocytes. Mechanistically, CCN1 enables FasL-induced apoptosis in cardiomyocytes through dismantling caspase inhibitor XIAP [13]. Because CCN1 is induced by DOX, XIAP may be suppressed by DOX via CCN1. Indeed, the levels of XIAP were reduced after DOX treatment in nontransfected or NTG-cells (Figure 4A, 4D). By contrast, the levels of XIAP were not reduced by DOX in siCCN1-cells, thereby hindering the activation of caspase-3 (Figure 4A, 4C, 4D).

### Disrupting CCN1-α_6_β_1_ engagement by T1 peptide ameliorates DOX cardiotoxicity

**Figure 5:**
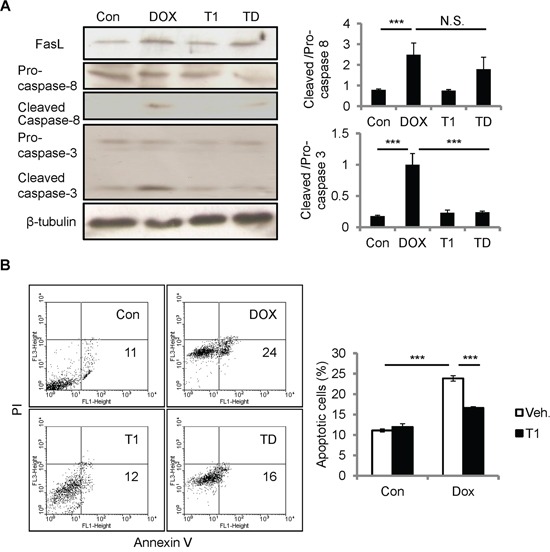
Disrupting CCN1/α_6_β_1_ engagement inhibited DOX-induced apoptosis but not FasL induction or caspase-8 activation in H9c2 cells **A, B.** Where indicated, cells were pre-incubated with T1 peptide (20 μM) for 1 h prior to DOX treatment (10 μM, 5 h). (A) Total cell lysates were immunoblotted with anti-caspase-3, anti-caspase-8, or anti-β-tubulin antibodies. Protein bands were quantified and expressed as in Figure 2. (B) Apoptosis was assessed by annexin V/PI staining using flow cytometry. The percentage of apoptotic cells (annexin V^+^/PI^−^) was scored from the lower right quadrant of the histogram. Data are expressed as mean ± SEM of triplicate experiments.

The resistance to DOX cardiotoxicity in *Ccn1^dm/dm^* mice suggests the involvement of integrin α_6_β_1_ in CCN1 signaling. To investigate whether α_6_β_1_ is necessary for facilitating apoptosis induced by DOX, we used T1 peptide to block the binding between CCN1 and α_6_β_1_. T1, a 17-residue peptide sequence derived from an α_6_β_1_-binding site of CCN1, specifically antagonizes integrin α_6_β_1_ [22]. Though DOX can be absorbed by cells and works intracellularly, the incubation with T1 peptide (20 μM) in the media 1 h prior to the addition of DOX (10 μM; 5 h) completely blunted caspase-3 activation in H9c2 cells (Figure 5A), conceivably through blocking the effect of CCN1. Consistently, DOX-induced apoptosis was repressed by T1 peptide (Figure 5B). Apoptosis was assessed using flow cytometry analysis after annexin V/propidium iodide (PI) double staining. Notably, the increase of FasL and activation of caspase-8 (cleaved caspase-8) by DOX were not affected by T1 peptide (Figure 5A), suggesting that CCN1 is not involved in the induction of FasL by DOX.

### T1 peptide partially reduces ROS accumulation by DOX

**Figure 6:**
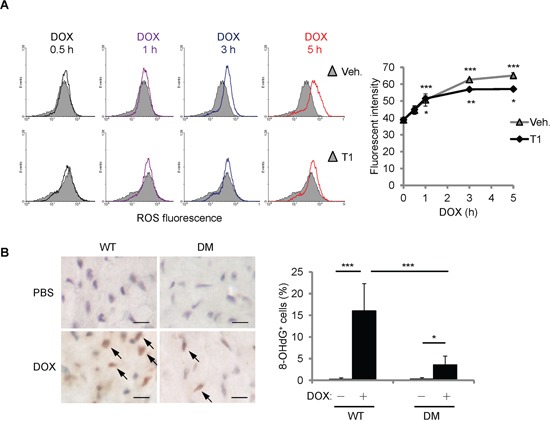
DOX increased cellular ROS partially independent of CCN1 **A.** Where indicated, H9c2 cells were pretreated with T1 peptide (20 μM) for 1 h before being treated with DOX (10 μM) for the indicated times. Fluorescent ROS indicator CM-H_2_DCFDA was delivered to the cells 15 min prior to the end of DOX treatment. The level of ROS was measured using flow cytometry. Cells with no DOX treatment were used as a control for basal ROS levels (grey area in the histogram overlays). Quantified ROS fluorescent intensities were expressed as means ± SEM from triplicate experiments. The statistical significance was compared between experimental and control (0 h) groups. **B.** The cardiac tissue from WT or *Ccn1^dm/dm^* mice 14 days after PBS control or DOX treatments was immunostained with anti-8-OHdG antibody (brown; arrows). The percentages of 8-OHdG^+^ cells were determined from 10 random high-power views per tissue section and 4 sections per mouse (n = 6 for each group), and presented as means ± SEM. Bars: 10 μm.

To further understand how T1 peptide affects the cellular responses to DOX, we first examined the levels of ROS accumulation after DOX treatment. H9c2 cells were treated with DOX (10 μM) and/or T1 peptide (20 μM) for the time indicated, and then fluorescent ROS indicator CM-H_2_DCFDA was administered 15 min prior to the end of treatment. ROS fluorescence was measured using flow cytometry. Cellular ROS accumulation induced by DOX was not affected by the combination of T1 peptide within 1 h of treatment (Figure 6A). Interestingly, while the elevation of ROS levels by DOX continued until 5 h of treatments, the levels of ROS reached a plateau after 1 h of T1+DOX treatment (Figure 6A), suggesting that the initial ROS induction by DOX does not require CCN1. The extension of ROS increase beyond 1 h of DOX treatment can be attributed to the newly synthesized CCN1 protein. To assess the oxidative stress induced by DOX *in vivo*, we examined the mouse hearts 14 days after DOX treatment by immunohistochemical staining with anti-8-OHdG antibody, a marker of oxidative DNA damage [23]. No detectable 8-OHdG^+^ cells were found in the mice receiving PBS. 15% of cardiomyocytes in DOX-treated WT mice were 8-OHdG^+^ (Figure 6B, brown nuclei indicated by arrows). ~3% of the cardiomyocytes in DOX-treated *Ccn1^dm/dm^* mice were 8-OHdG^+^ (Figure 6B). Together, these results demonstrated that DOX can generate oxidative stress independent of CCN1.

### DOX diminishes anti-apoptotic XIAP through CCN1 signaling

**Figure 7:**
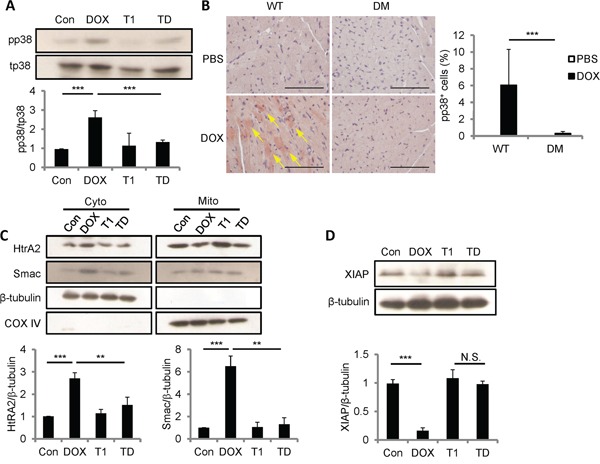
DOX promoted p38 activation, Smac and HtrA2 translocation to cytosol, and XIAP reduction through CCN1/α_6_β_1_ signaling **A, C, D.** H9c2 Cells were pretreated with or no T1 peptide (20 μM) for 1 h before being treated with or no DOX (10 μM, 5 h). Total cell lysates were immunoblotted with anti-total (t)p38 or anti-phospho (p)p38 antibodies (A); or with anti-XIAP and anti-β-tubulin antibodies (D). Cytosolic and mitochondrial fractional lysates were immunoblotted with anti-HtrA2, anti-Smac, anti-β-tubulin, or anti-COX IV antibodies (C). Protein band intensity was quantified and expressed as in Figure 2 from 3 independent experiments. **B.** Cardiac tissue from WT or *Ccn1^dm/dm^* mice 14 days after PBS or DOX treatment was immunostained with anti-pp38 antibody (reddish-brown; arrows). The percentages of pp38^+^ cells were determined from 10 random high-power views per tissue section and 4 sections per mouse (n = 6 for each group), and presented as means ± SEM. Bars: 100 μm.

ROS generated by CCN1 leads to the activation of p38 MAPK and following mitochondrial pathways in H9c2 cells [13]. We investigated whether p38 MAPK activation is regulated by DOX by Western blotting with antibodies specifically against phosphorylated (active) p38 (pp38) or total p38 (tp38). Pp38 was significantly increased by DOX (10 μM; 5 h) (Figure 7A). The activation of p38 by DOX was completely blocked by the combination of T1 peptide (20 μM; 1-h pretreatment), suggesting that CCN1 mediates the activation of p38 by DOX. To confirm the activation of p38 *in vivo*, heart tissue from DOX-treated WT or *Ccn1^dm/dm^* mice was immunohistochemically stained with anti-pp38 antibody. Pp38^+^ cardiomyocytes were markedly increased after DOX treatment in WT mice (reddish-brown staining in Figure 7B; arrows), but were not found in *Ccn1^dm/dm^* mice. We next examined the release of mitochondrial Smac and HtrA2, a downstream event after p38 activation [13], by immunoblotting the fractional cell lysates after DOX treatment. The levels of HtrA2 and Smac were elevated in the cytosolic fractions of H9c2 cells by DOX (Figure 7C). The translocation of HtrA2 and Smac induced by DOX was blunted by T1 peptide (Figure 7C). COX-IV served as a mitochondrial contamination control. Smac promotes apoptosis through binding to XIAP and neutralizing its anti-apoptotic activity [24]. HtrA2, a serine protease, diminishes XIAP through catalytic cleavage [25]. Indeed, the levels of XIAP were lowered after DOX treatment, and were not reduced in DOX+T1-treated cells (Figure 7D). Consistently, DOX failed to lower XIAP levels in CCN1-knockdown (siCCN1) cells (Figure 4A).

## DISCUSSION

**Figure 8:**
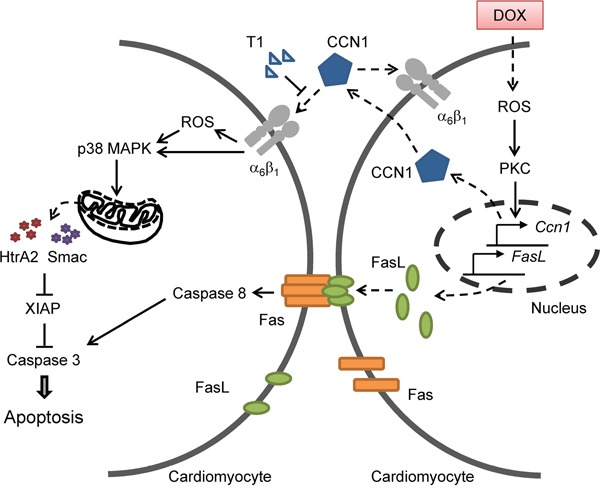
A diagram illustrates the mechanism by which CCN1 mediates DOX cardiotoxicity through autocrine and paracrine signaling by engaging integrin α_6_β_1_ to elicit signaling to dismantle the inhibition of XIAP and facilitate apoptosis

The clinical use of DOX is limited by its cardiotoxicity. Here we showed that matricellular protein CCN1 mediates the cardiotoxicity of DOX. CCN1 enables FasL-induced apoptosis in cardiomyocytes. We found that CCN1 and FasL were upregulated by DOX in the mouse heart or in cultured cardiomyocytes. Knockin mice expressing an α_6_β_1_-binding defective CCN1 mutant (CCN1-DM) became resistant to DOX-induced cardiomyopathy. In cultured cardiomyoblasts, CCN1 mediated the reduction of XIAP by DOX to facilitate apoptosis through activating p38 MAPK and the release of Smac and HtrA2 from mitochondria. Disrupting the binding between CCN1 and α_6_β_1_ by T1 peptide effectively abolished apoptosis induced by DOX (Figure 8).

As a matricellular protein, CCN1 does not induce mitogenesis or apoptosis on its own but acts through enhancing other mitogenic or apoptotic factors [14]. Thus, the physiological roles of CCN1 depend not only on the expression profile of target-cell-surface integrin receptors but on the environmental cues as well. Here we found that both CCN1 and FasL were induced in the myocardium of mice treated with DOX. DOX directly upregulated CCN1 and FasL expression to promote apoptosis in cultured H9c2 cells. *Ccn1^dm/dm^* mice were resistant to DOX-induced cardiomyopathy despite the persistent induction of FasL, demonstrating the essential role of CCN1/α_6_β_1_ signaling in DOX cardiotoxicity. Consistently, T1 peptide prevented DOX-induced apoptosis in H9c2 cells by maintaining XIAP protein levels to inhibit caspase-3 activation, while the induction of FasL by DOX remained. Notably, higher FasL induction was observed in the myocardium of WT than of *Ccn1^dm/dm^* mice after DOX treatment. Because the tissue was examined 14 days after DOX injection, higher FasL expression possibly was induced by the cardiac injury and inflammation developed in WT mice.

Though apoptosis induced by DOX was completely abrogated by T1 peptide in H9c2 cells, DOX-induced ROS was only partially reduced (Figure 6A), suggesting that DOX can increase ROS independent of CCN1. Because ROS are required for the induction of both CCN1 and FasL, the initial ROS elevation directly by DOX is responsible for the induction of CCN1 and FasL and will not be blocked by T1 peptide (Figure 8). The CCN1-independent ROS elevation by DOX can activate PKC to induce the expression of CCN1, which further increases ROS through 5-lipoxygenase and NADPH oxidases after engaging integrin α_6_β_1_ [13]. Consistently, 8-OHdG^+^ cells were increased even in *Ccn1^dm/dm^* mice. The overly increased numbers of 8-OHdG^+^ cells in WT mice may be caused by a combination of direct effects from DOX and secondary effects from tissue injury and inflammation. Though ROS can be induced by both DOX (independent of CCN1) and CCN1, CCN1/α_6_β_1_ evidently triggers a distinct set of events that cannot be replaced by ROS alone. A potential candidate is integrin-linked kinase (ILK), which binds to the cytoplasmic tail of β_1_ and β_3_ integrins [26]. ILK interacts with β1 integrins to promote p38 MAPK activation and cell cycle arrest of epithelial cells in renal tubulogenesis [27]. It is tempting to speculate that ILK is involved in DOX cardiotoxicity through transducing CCN1/α_6_β_1_ signaling. This CCN1-α_6_β_1_-ILK-p38 signaling axis underlying DOX cardiotoxicity is distinct from the mechanism of the anticancer activity of DOX, and serves as a more specific target for chemoprotection.

To establish CCN1 as a chemoprotective target, the role of CCN1 in tumorigenesis needs to be considered. CCN1 overexpression is associated with the progression of various tumors, including prostate cancer, breast cancer, glioma, colorectal cancer, and osteosarcoma, through its ability to promote angiogenesis, cell migration or proliferation [28, 29]. In contrast to in cardiomyocytes, overexpression of CCN1in human breast cancer MCF-7 cells upregulates XIAP expression to promote chemoresistance to paclitaxel, Adriamycin, and β-lapachone through integrin α_v_β_3_/α_v_β_5_ and NFκB signaling pathway [30]. Thus, blocking CCN1/α_6_β_1_ signaling is not expected to impose any negative impact to patients receiving DOX treatment. However, CCN1 can also serve as a tumor suppressor for endometrial and lung cancers [31, 32]. Moreover, despite conflicting reports regarding the role of CCN1 in hepatocarcinogenesis [33, 34], CCN1 has been suggested to suppress hepatocellular carcinoma by inhibiting carcinogen-induced compensatory hepatocyte proliferation through integrin α_6_β_1_ [35]. The risks of latent tumorigenesis resulting from disrupting CCN1/α_6_β_1_ signaling long term need to be further evaluated. Nevertheless, because replicating cells are more sensitive to DOX [2], cotreatment of DOX likely will compensate the loss of the antineoplastic activities from disrupting CCN1/α_6_β_1_ signaling in hepatocytes.

In summary, we identified matricellular protein CCN1 as a novel downstream effector for DOX cardiotoxicity. CCN1 is induced by DOX in cultured cardiomyoblast H9c2 cells and in mouse hearts to promote cell death and cardiac dysfunction. CCN1 acts through engaging integrin α_6_β_1_ to trigger the activation of p38 MAPK, translocation of mitochondrial Smac and HtrA2, thereby diminishing anti-apoptotic XIAP and facilitating apoptosis in cardiomyocytes. Disrupting CCN1/α_6_β_1_ engagement prevents DOX-induced cardiomyopathy in mice.

## MATERIALS AND METHODS

### Mouse model of DOX-induced cardiomyopathy

All animal experiments conformed to the guidelines of the Institutional Animal Care and Use Committee of the National Chen Kung University. Two knockin transgenic mouse lines—*Ccn1^+/lacZ^* and *Ccn1^dm/dm^*—on the C57BL/6 background were obtained from Lester Lau, PhD (University of Illinois at Chicago, IL). Cardiomyopathy was induced in mice of 3- to 6-month-old males by a single injection of DOX (15 mg/kg; i.p.) (Sigma), as previously described [36]. Mice were euthanized by cervical dislocation before the hearts were collected from *Ccn1^+/lacZ^* 1 day after DOX treatment, or from WT and *Ccn1^dm/dm^* mice 14 days after DOX for histological analysis (n=6 for each group).

### Histology

For CCN1 expression, 14 μm cryo sections of heart tissue from *Ccn1^+/lacZ^* mice were stained with X-gal as previously described [16]. For histological analysis, 8 μm paraffin sections of heart tissue from WT or *Ccn1^dm/dm^* mice were subjected to Masson's trichrome (Sigma) staining or TUNEL staining (Millipore) following standard procedures. For immunohistochemical staining, paraffin tissue sections were antigen-retrieved before being incubated with primary antibodies, then secondary antibodies, then visualized with AEC detection IHC kit (Abcam) or M.O.M. Immunodetection kit (Vector Laboratories). Primary antibodies used include anti-FasL (Abcam 15285, 1:100), anti-8-OHdG (JaICA, MOG-020P, 1:100), and anti-phospho-p38 (pp38) (Cell Signaling 4631, 1:50).

### Electrocardiography and echocardiography

Electrocardiograms were recorded using a PowerLab 8/30 (ADInstruments) on mice (n=4) anesthetized with chloral hydrate (300 mg/kg; i.p.) in the supine position. Echocardiography was performed using a Vevo 770 (Visual Sonics) on mice (n=4) anesthetized with 2% isoflurane. Left ventricular ejection fraction (EF) and fractional shortening (FS) were computed from echocardiographic measurements by the Vevo software.

### Cell culture

Rat cardiomyoblast H9c2 cells were purchased from American Type Culture Collection and maintained at 37°C in Dulbecco's Modified Eagle's Medium with 10% fetal bovine serum (Invitrogen). H9c2 cells were serum starved for overnight before treated with 10 μM DOX and/or 20 μM T1 peptide (Angene Biotech, Taiwan) for the time indicated in the figure legends. To block PKC, cells were pretreated with 1 μM calphostin C (Sigma) for 1 h.

### RNA interference

H9c2 cells were transfected with 100 nM *Ccn1* siRNA or a non-targeting control (Invitrogen) using Lipofectamine 3000 (Invitrogen). Cells after transfection were incubated for 2 days before following treatments. *Ccn1* siRNA sequences: 5′-GCA GCA AGA CCA AGA AAT CC-3′; 5′-TTC TGG TCT GCA GAG GTG TG-3′.

### Western blotting

Cytoplasmic and mitochondrial fractional extracts were isolated using Mitochondria/Cytosol Fractionation Kit (BioVision). Total cell lysates or fractional extracts were electrophoresed, transblotted, and immunoblotted following standard procedures. Primary antibodies used include anti-FasL (Santa Cruz sc6237, 1:2000), anti-CCN1 (1:5000) (a generous gift from Shr-Jeng Leu, PhD; National Yang Ming University, Taiwan), anti-XIAP (R&D AF8221, 1:4000), anti-Caspase-3 (Cell Signaling 9662, 1:4000), anti-Caspase-8 (Cell Signaling 9429, 1:1000), anti-HtrA2 (R&D AF1458, 1:4000), anti-Smac (R&D AF7891, 1:1000), anti-pp38 (Cell Signaling 9211, 1:4000), anti-tp38 (Cell Signaling 9212, 1:4000), anti-COX IV (Cell Signaling 4844, 1:1000), and anti-β-tubulin (Abcam ab6046, 1:8000). General reagents and chemicals were from Sigma.

### RNA isolation and qRT-PCR

Total RNA was extracted from H9c2 cells using TRIzol Reagent (Invitrogen). RT-PCR was carried out using the iScript cDNA synthesis kit (Bio-Rad) following the manufacturer's protocol. qRT-PCR was performed using SYBR Green-based (Invitrogen) StepOnePlus™ Real-Time PCR System. Primer pairs were: *Ccn1* forward primer: 5′-GCA GCA AGA CCA AGA AAT CC-3′, reverse primer: 5′-TTC TGG TCT GCA GAG GTG TG-3′; *FasL* forward primer: 5′-AAA GAC CAC AAG GTC CAA CA-3′, reverse primer: 5′-AGT CTC TAG CTT ATC CAT GA-3′; and Cyclophilin forward primer: 5′-GGC AAA TGC TGG ACC AAA CAC-3′, reverse primer: 5′-TTC CTG GAC CCA AAA CGC TC-3′.

### Annexin V/PI staining

Treated H9c2 Cells were harvested and incubated with FITC-conjugated annexin V antibody (BD Biosciences) and PI at room temperature for 20 min before they were analyzed using flow cytometry. The results were further analyzed using WinMDI software.

### ROS measurements

Live cells were loaded with ROS dye CM-H_2_DCFDA (5 μM) (Sigma) 15 min prior to the end of DOX treatment. Subsequently, the cells were harvested and resuspended in cold PBS containing 5% FBS. Fluorescence was measured using flow cytometry and analyzed with WinMDI.

### Statistical analysis

All assays were repeated at least 3 times and yielded similar patterns. Data are mean ± SEM. Statistical analysis was carried out using one-way (cell-based assays) or two-way (animal studies) ANOVA and *post hoc* tests. Significance was set at *p*<0.05 and indicated as N.S.: no significant difference; *: *p*<0.05; **: *p*<0.01; or ***: *p*<0.001.
